# Whole-Genome Selective Scans Detect Genes Associated With Important Phenotypic Traits in Sheep (Ovis aries*)*

**DOI:** 10.3389/fgene.2021.738879

**Published:** 2021-11-18

**Authors:** Song-Song Xu, Lei Gao, Min Shen, Fenghua Lyu

**Affiliations:** ^1^ College of Animal Science and Technology, China Agricultural University, Beijing, China; ^2^ Guangdong Laboratory of Lingnan Modern Agriculture, Genome Analysis Laboratory of the Ministry of Agriculture and Rural Affairs, Shenzhen Branch, Agricultural Genomics Institute at Shenzhen, Chinese Academy of Agricultural Sciences, Shenzhen, China; ^3^ State Key Laboratory of Sheep Genetic Improvement and Healthy Breeding, Xinjiang Academy of Agricultural and Reclamation Sciences, Shihezi, China

**Keywords:** artificial selection, genome-wide SNPs, sheep, phenotypic traits, genetic improvement

## Abstract

Sheep (*Ovis aries*) is one of the important livestock with diverse phenotypic traits. However, little is known about the molecular mechanism of diverse phenotypic traits in domestic sheep. Using the genome-wide high-density SNP data (600K) in 253 samples from 13 populations, we conducted the tests of selective sweeps (i.e., pairwise *F*_ST_ and XP-CLR) associated with several important phenotypic traits (e.g., tail types, horn morphology, prolificacy, coat pigmentation, ear size, milk production, meat production, body size and wool fineness). We identified strong selective signatures in previously reported (e.g., *T*, *RXFP2*, *BMPR1B*, *TYRP1*, *MSRB3*, *TF*, *CEBPA*, *GPR21* and *HOXC8*) and novel genes associated with the traits, such as *CERS6*, *BTG1*, *RYR3*, *SLC6A4*, *NNAT* and *OGT* for fat deposition in the tails, *FOXO4* for fertility, *PTCH1* and *EMX2* for ear size, and *RMI1* and *SCD5* for body size. Further gene annotation analysis showed that these genes were identified to be the most probable genes accounting for the diverse phenotypic traits. Our results provide novel insights into the genetic mechanisms underlying the traits and also new genetic markers for genetic improvement in sheep and other livestock.

## Introduction

Sheep (*Ovis aries*) is an excellent model species for investigating the genetic basis of diverse phenotypic traits under the effects of genetic drift, natural and artificial selection factors (Cao et al., 2020). Following domestication, as many as 1,400 breeds have been developed in sheep (Scherf, 2000). In particular, human-imposed selection has affected the species greatly over the past hundreds of years, and, thus, diverse phenotypic traits have been formed in different breeds, such as fat-rumped sheep (Kazakh Edilbai), thin-tailed sheep (Celle Black), Polled (Merino), high prolificacy sheep (Hu) and dairy sheep (Lacaune) (Wei et al., 2015; see Table 1).

**TABLE 1:** Summary information of 13 breeds of domestic sheep.

Breed origin	Breed name	Code	No. of samples	Sex	Phenotypic characteristics	Geographic origins	
						Latitude (°N)	Longitude (°E)
China	Celle Black Sheep	CLS	15	Female	Short thin-tailed and black or gray coat color	34.02	82.66
China	Tan sheep	TAN	15	Female	White coat color and seasonal reproductive	37.75	106.41
China	Hu sheep	HUS	15	Female	High prolificacy	32.44	120.25
Kazakhstan	Kazakh Edilbai	KAZ	9	Female	Fat-rumped and a wide and deep body	52.32	77.03
Afghanistan	Jill Wagner sheep	WGJ	11	Male/Female	Exceptionally large and floppy ears	37.13	79.93
Scotland	Shetland	SHL	11	Male/Female	Medium length legs and finely boned	51.17	4.20
Australia	Merino	MER	36	Male	Two horns	−25.27	133.78
Australia	Polled Merino	PME	19	Male	Hornlessness	−25.27	133.78
France	Meat Lacaune	LAM	34	Female	Meat type	43.97	2.99
France	Dairy Lacaune	LAC	36	Female	Dairy type	43.97	2.99
Germany	East Friesian sheep	EFR	22	Female	Dairy type	49.82	15.47
Russia	Caucasian	CAU	15	Female	Delicate wool	45.71	42.88
Italy	Altamurana	ALT	15	Female	Semi-fine wool	41.12	16.87

The recent availability of genome-wide SNPs gave a new momentum to identify the genetic variants underlying phenotypic traits (Kijas et al., 2012; Xu and Li 2017; Gui et al., 2020; Abousoliman et al., 2021; Zhou et al., 2021). Previous studies have identified a number of candidate genes or variants associated with meat, growth, milk, wool, reproduction, horns and tails in sheep, most of which have employed the low-density SNPs (50K) (see the review in Xu and Li, 2017) or whole-genome sequences (Li et al., 2020). However, to date, little is known regarding the molecular mechanism of diverse phenotypic traits in sheep, such as dairy and horn traits within breeds. Here, we applied genome-wide selective scans to detect critical genes associated with the phenotypic traits based on the Ovine Infinium HD BeadChip.

## Materials and Methods

### Genotypic and Phenotypic Data

**FIGURE 1:**
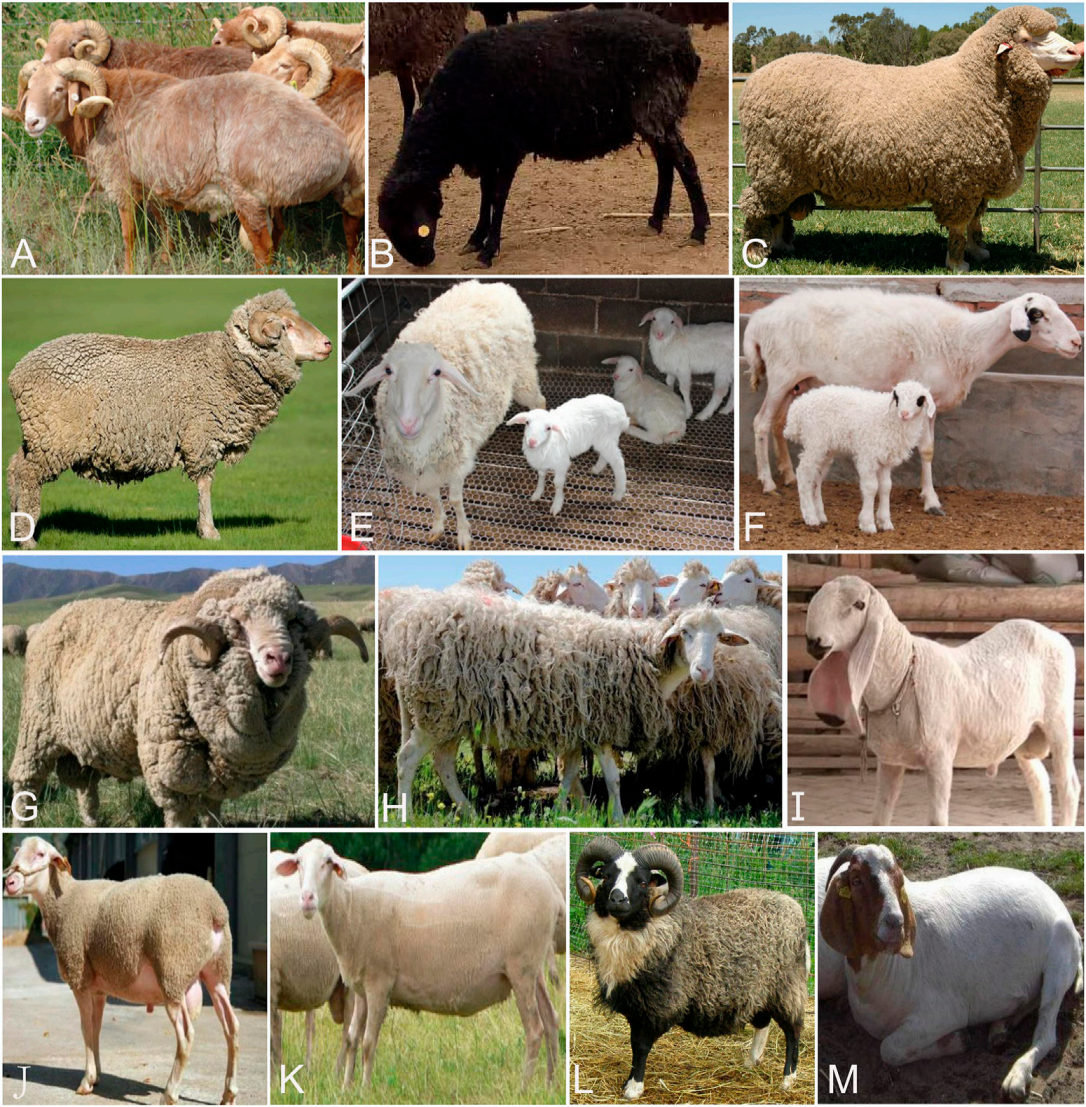
Images of domestic sheep **(A)** Kazakh Edilbai sheep **(B)** Celle Black sheep **(C)** Polled Merino **(D)** Merino **(E)** Hu sheep **(F)** Tan sheep **(G)** Caucasian sheep **(H)** Altamurana sheep **(I)** Jill Wagner sheep **(J)** Meat Lacaune **(K)** Dairy Lacaune **(L)** Shetland **(M)** East Friesian sheep. Photo credits are showed in supplementary table 2.

We collected 253 individuals from 13 domestic sheep populations with typical phenotypic traits to investigate the genetic variants under long-term artificial selection (Figure 1 and Table 1; Kijas et al., 2014; Xu et al., 2017; Zhao et al., 2017; Gao et al., 2018; Xu et al., 2018; Rochus et al., 2018; Cao et al., 2020). Whole genome SNP datasets (Ovine Infinium HD SNP BeadChip) of these individuals were obtained from previous studies (Xu et al., 2017; Zhao et al., 2017; Gao et al., 2018; Xu et al., 2018) and divided into 9 pairs populations (Table 2). We performed two different selection methods, the pairwise *F*_ST_ (Weir and Cockerham, 1984) and the cross-population composite likelihood ratio (XP-CLR) test (Chen et al., 2010). The identification of common signatures by the different algorithms and assumptions might be seen as good reliability of the results while reducing the likelihood of false positives.

**TABLE 2:** Putative genes under selection based on pairwise *F*_ST_ and XP-CLR.

Traits	Populations	Functional genes
Tail shapes	Kazakh Edilbai sheep vs Celle Black sheep	*CERS6, BTG1, RYR3, T, SLC6A4, NNAT, OGT*
Horn morphology	Merino sheep vs Polled Merino sheep	*RXFP2*
Fertility	Hu sheep vs Tan sheep	*BMPR1B, FOXO4*
Coat-color pigmentation	Celle Black sheep vs Tan sheep	*TYRP1, KIT*
Wool fineness	Caucasian sheep vs Altamurana sheep	*HOXC8, HOXC12, HOXC13, MSI2, DSG1*
Ear size	Jill Wagner sheep vs Kazakh Edilbai sheep	*PTCH1, MSRB3, EMX2*
Meat production	Meat Lacaune sheep vs Dairy Lacaune sheep	*CEBPA, CEBPG, DLX3, DLX4, GBAS, NSMAF, PDE3A, PEPD, SDCBP, TNRC6A, UTRN*
Body size	Kazakh Edilbai sheep vs Shetland sheep	*RMI1, GPR21, SCD5, CADM1*
Milk fat yield	East Friesian sheep vs Caucasian sheep	*TF*

### SNP Data Quality Control

We implemented strict quality control of the SNP dataset using the PLINK v.1.09 software (Purcell et al., 2007). We removed individuals and SNPs that met any of the following criteria: 1) SNPs without chromosomal or physical locations; 2) SNPs with >0.02 missing data; 3) individuals with a genotyping rate <0.95; 4) minor allele frequency (MAF) < 0.05; and 5) the *p*-value of Fisher’s exact test for Hardy-Weinberg equilibrium (HWE) < 0.00001. Consequently, the final data after filtering contained various sets of SNPs and individuals in the comparison tests, such as 506,350 SNPs and 24 individuals (9 Kazakh Edilbai sheep *vs* 15 Celle Black Sheep) for the trait of tail shape, 485,747 SNPs and 55 individuals (36 Merino sheep *vs* 19 Polled Merino sheep) for horn morphology, 514,795 SNPs and 30 individuals (15 Hu sheep *vs* 15 Tan sheep) for fertility, 509,580 SNPs and 30 individuals (15 Celle Black Sheep *vs* 15 Tan sheep) for coat-color pigmentation, 529,338 SNPs and 30 individuals (15 Caucasian sheep *vs* 15 Altamurana sheep) for wool fineness, 519,650 SNPs and 20 individuals (11 Jill Wagner sheep *vs* 9 Kazakh Edilbai sheep) for ear size, 483,150 SNPs and 70 individuals (34 Meat Lacaune sheep *vs* 36 Dairy Lacaune sheep) for meat production, 528,576 SNPs and 20 individuals (9 Kazakh Edilbai sheep *vs* 11 Shetland sheep) for body size, 471,257 SNPs and 31 individuals (16 East Friesian sheep *vs* 15 Caucasian sheep) for milk production (Supplementary Table S1).

### Genomic Selection Signals Analysis

To identify the genomic signatures of selection between pairwise populations of contrasting these phenotypes in domestic sheep, we calculated the *F*_ST_ values (Weir and Cockerham, 1984) for each SNP using the program Genepop v4.2 (Rousset, 2008). We took the top 0.02% of the empirical distribution of *F*_ST_ as the putative selective signals. Further, we calculated the XP-CLR scores for the 200 bp intervals along the chromosomes using the parameters (“-w1 0.005200 2000-*p* 0 0.95”). For each chromosome, we averaged the XP-CLR scores per window across non-overlapping 10 kb windows. We selected the top 0.05% of these windows as the putative selective regions.

## Results and Discussion

**FIGURE 2:**
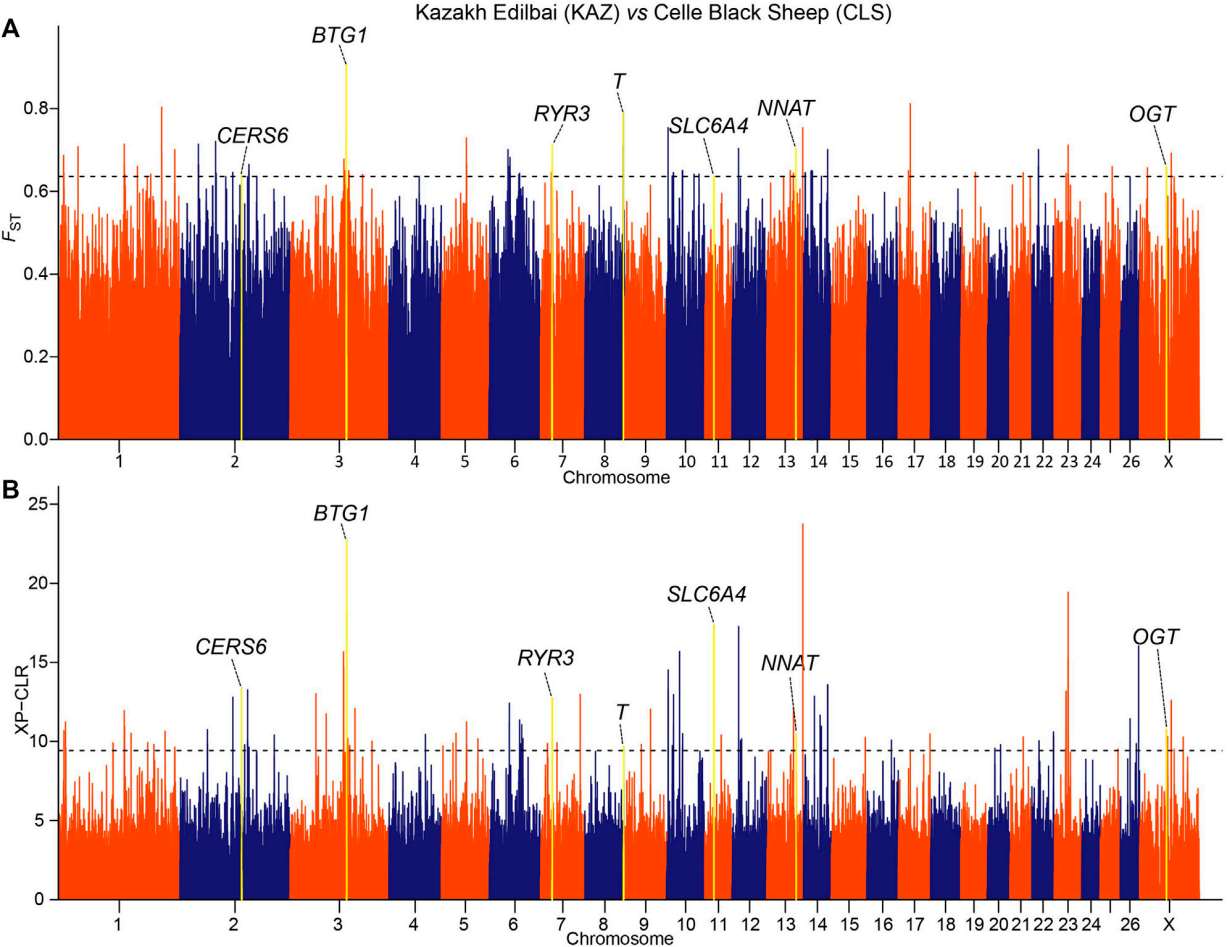
Manhattan plots of **(A)** pairwise *F*_ST_ and **(B)** XP-CLR selection tests with tail shapes in the comparison of Kazakh Edilbai (KAZ) and Celle Black Sheep (CLS) populations. The top 0.02% of the empirical distribution of *F*_ST_ and 0.05% of the XP-CLR scores are indicated by dotted lines, respectively.

**FIGURE 3:**
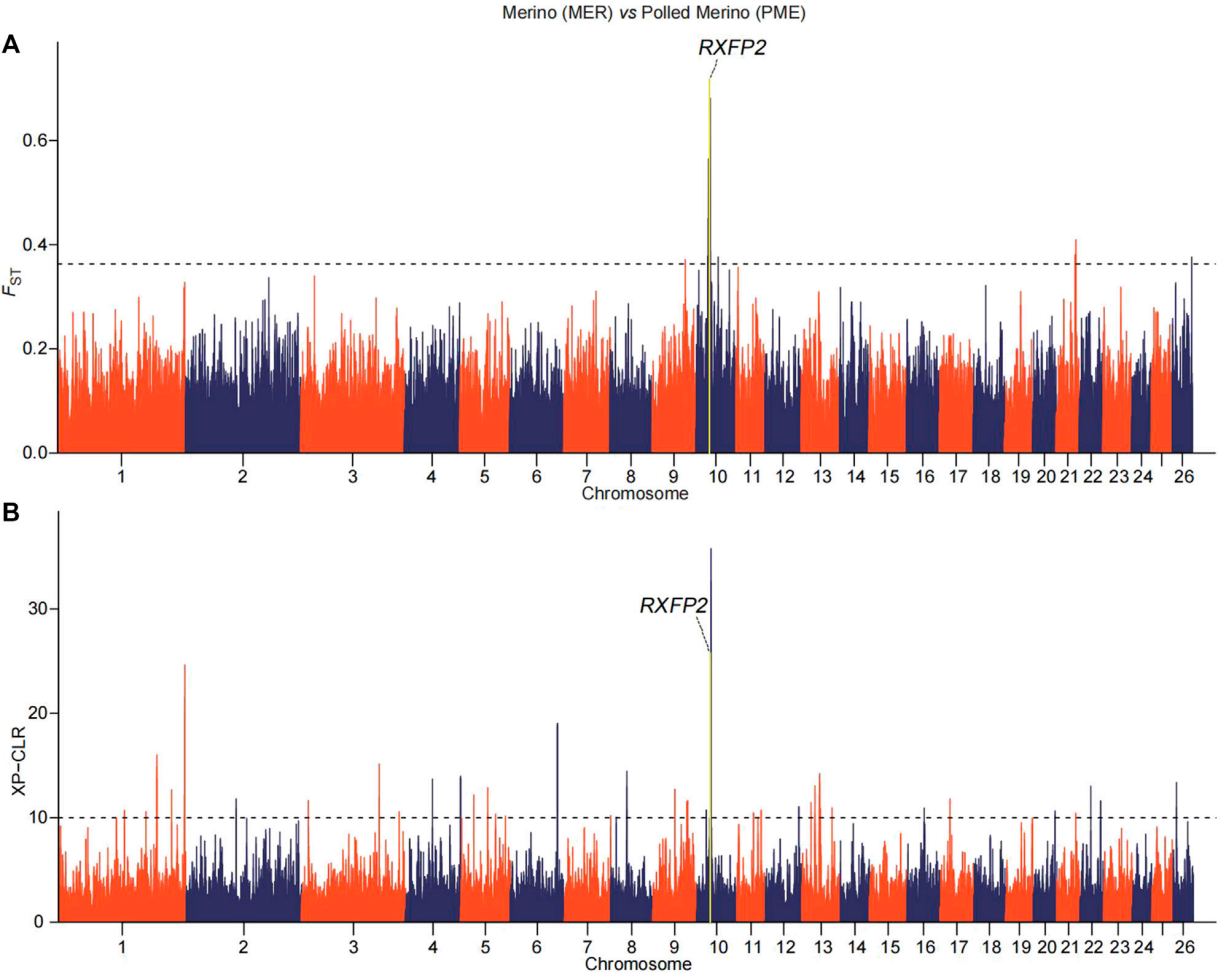
Manhattan plots of **(A)** pairwise *F*_ST_ and **(B)** XP-CLR with horn morphology in the comparison of Polled Merino (PME) and Merino (MER) breeds. The top 0.02% of the empirical distribution of *F*_ST_ and 0.05% of the XP-CLR scores are indicated by dotted lines, respectively.

We implemented selection screening in 9 pairs of populations: KAZ and CLS for tail types, PME and MER for presence or absence of horn, HUS and TAN for fertility, CLS and TAN for coat colors, ALT and CAU for wool fineness, WGJ and KAZ for ear size, LAC and LAM for meat types, KAZ and SHL for body size and EFR and CAU for milk production. We detected significant common signals located within or neighboring both novel and previously reported functional genes. A total of 36 genes were shared between the two selection scan metrics (Table 2). For example, seven genes (*CERS6*, *BTG1*, *RYR3*, *T*, *SLC6A4*, *NNAT* and *OGT*) (Figure 2 and Supplementary Tables 3, 4) were identified to be associated with different tail shapes (*i.e.*, fat-tailed *vs* thin-tailed) (Joo and Yun, 2011; Tsai et al., 2013; Ruan et al., 2014; Xiao et al., 2016; Dias et al., 2016; Turner et al., 2018; Zhi et al., 2018). In the horned *vs* polled sheep, the well-known horn morphology-associated gene *RXFP2* has been implicated as a strong candidate gene that explains the presence or absence of horn in sheep (Figure 3 and Supplementary Tables S5, 6; Hu et al., 2019). In the high prolificacy *vs* low prolificacy breeds, the gene *BMPR1B* could be involved in the variation in litter size of females (Supplementary Figure S1 and Supplementary Tables S7, 8; Rossetti et al., 2017). In addition, the two genes *TYRP1* and *KIT* had been directly implicated in the mechanism of coat-colour pigmentation in the white *vs* non-white coat-colour breeds (Supplementary Figure S2 and Supplementary Tables S9, 10; Vage et al., 2003). The five genes *MSI2*, *DSG1*, *HOXC8*, *HOXC12* and *HOXC13* were crucial regulators for wool fineness in the fine-wool *vs* semi-fine wool sheep (Supplementary Figure S3 and Supplementary Tables S11, 12; Awgulewitsch, 2003). The genes *MSRB3*, *PTCH1* and *EMX2* were functionally associated with ear size in the large and floppy *vs* normal ears sheep (Supplementary Figure S4 and Supplementary Tables S13, 14; Rhodes et al., 2003; Wei et al., 2015; Shin et al., 2017). The eleven genes (*CEBPA, CEBPG, DLX3, DLX4, GBAS, NSMAF, PDE3A, PEPD, SDCBP, TNRC6A* and *UTRN*) had been reported to be involved in regulating meat production such as intramuscular fat, drip loss, marbling score, meat traceability and longissimus muscle in the meat-type *vs* non-meat-type sheep (Supplementary Figure S5 and Supplementary Tables S15, 16; Lobbert et al., 1996; Nonneman et al., 2013; Ayuso et al., 2015). The genes *RMI1*, *GPR21*, *SCD5* and *CADM1* might play important roles in regulating embryo development, body weight, lipid metabolism and energy homeostasis involved in differences in body size (Supplementary Figure S6 and Supplementary Tables S17, 18; Guo et al., 2013). The gene *TF* was associated with milk production in the dairy-type *vs* non-dairy-type sheep (Supplementary Figure S7 and Supplementary Tables S19, 20; Ju et al., 2011).

In particular, we detected novel genes with functions associated with specific traits in sheep, such as *CERS6*, *BTG1*, *NNAT* and *OGT* for fat deposition in the tail of sheep, *FOXO4* for fertility, *PTCH1* and *EMX2* for ear size, and *SCD5* for body size. As a negative regulator of *β*-oxidation, the expression of *CERS6* was significantly increased in subcutaneous fat of obese subjects with type 2 diabetes (Raichur et al., 2019). The *BTG1* gene plays a key role in intramuscular fat deposition by regulating adipose-derived stem cell differentiation to osteocytes and myocytes (Moisa et al., 2015). The gene *NNAT*, as an endoplasmic reticulum proteolipid implicated in the intracellular signalling, is associated with severe obesity (Scott et al., 2013). The gene *OGT* is an important determinant of fatty acid synthesis in the mouse liver, which plays a critical role in fat deposition (Guinez et al., 2011; Kos et al., 2009). The *FOXO4* gene has an important role in the activity of corpus luteum that is linked to folliculogenesis (Pisarska et al., 2009). The *PTCH1* gene plays a critical role in the microcephaly, developmental delay, short stature, and facial dysmorphism by stimulating sonic hedgehog homolog (SHH) pathway (Derwinska et al., 2009). The *EMX2* gene is highly expressed in mouse inner ear, with the role of activating early hair cell development (Holley et al., 2010). The *SCD5* gene is linked to the regulator of sterol regulatory element-binding proteins involved in the development of body size (Baeza et al., 2013). Taken together, the apparent differences in the phenotypic traits among the breeds might be explained by diverse regulation mechanisms.

Noteworthy, we did not detect previously reported important functional genes associated with specific traits, for example, *PDGFD* and *BMP15*, which are associated with fat deposition in the tails of sheep (Li et al., 2020) and litter size (Xu et al., 2018), respectively. The main reason could be complex genetic mechanisms of phenotypic traits, for example, fertility was regulated by different major functional genes *BMP15*, *NCOA1* and *NF1* for Wadi, Icelandic and Finnsheep, respectively (Xu et al., 2018). In addition, we identified candidate functional genes different from those identified in earlier investigations, which could be due to that the power for general linear models to detect such associations will be weak when treating quantitative traits given the small sample size (Xu et al., 2018). Furthermore, these breeds could have been subjected to selection on specific traits (e.g., body weight) through environmental variables such as climate, diet and diseases. However, we did not obtain detailed information for these variables in our data analysis. Thus, these variables were not taken into account in our data analysis, which would be essential for future study.

In conclusion, we detected some novel and previously reported functional genes associated with particular phenotypic traits under strong and long-term artificial selection. Nevertheless, associations between these genes detected in two tests and the specific traits should be worthy of further exploration in future investigations. These findings contribute to understanding of the genomic consequences of artificial selection in the genomes of domestic sheep.

## Data Availability

The original contributions presented in the study are included in the article/Supplementary Material further inquiries can be directed to the corresponding author.
