# Supplementary material for: Whole-Genome Selective Scans Detect Genes Associated With Important Phenotypic Traits in Sheep (Ovis aries)
Source: Front Genet. 2021 Nov 18;12:738879. doi: 10.3389/fgene.2021.738879 (PMC8637624; doi:10.3389/fgene.2021.738879)
Supplement: Supplementary file 1 [file DataSheet7.PDF]

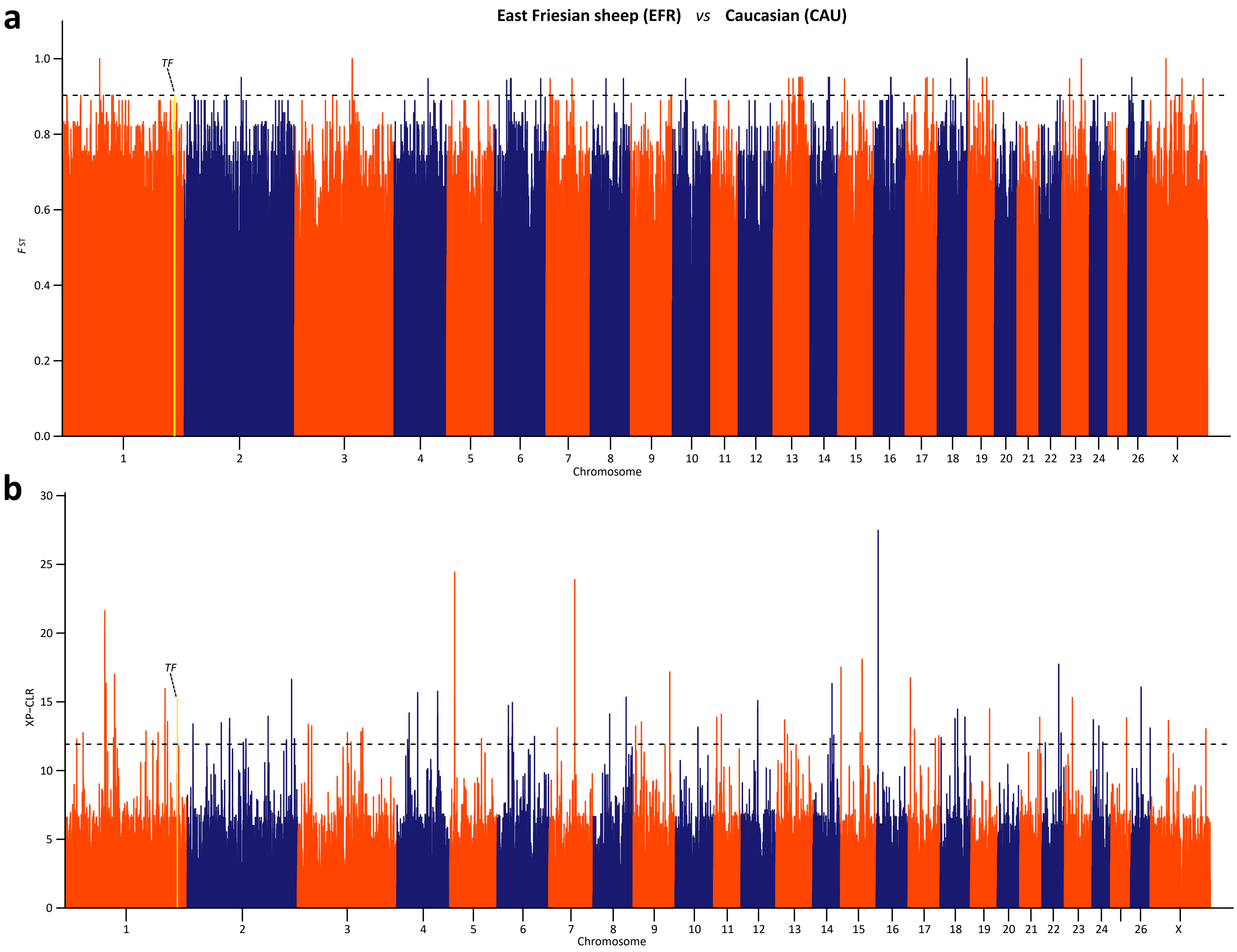

Supplementary Figure 9. Manhattan plots of **a**, pairwise F_{ST} and **b**, XP-CLR with milk fat yield in the comparison of East Friesian sheep (EFR) and Caucasian (CAU) breeds. The top 0.02% of the empirical distribution of F_{ST} and 0.05% of the XP-CLR scores is indicated by a dotted line.
