# Supplementary material for: Whole-Genome Selective Scans Detect Genes Associated With Important Phenotypic Traits in Sheep (Ovis aries)
Source: Front Genet. 2021 Nov 18;12:738879. doi: 10.3389/fgene.2021.738879 (PMC8637624; doi:10.3389/fgene.2021.738879)
Supplement: Supplementary file 7 [file DataSheet5.PDF]

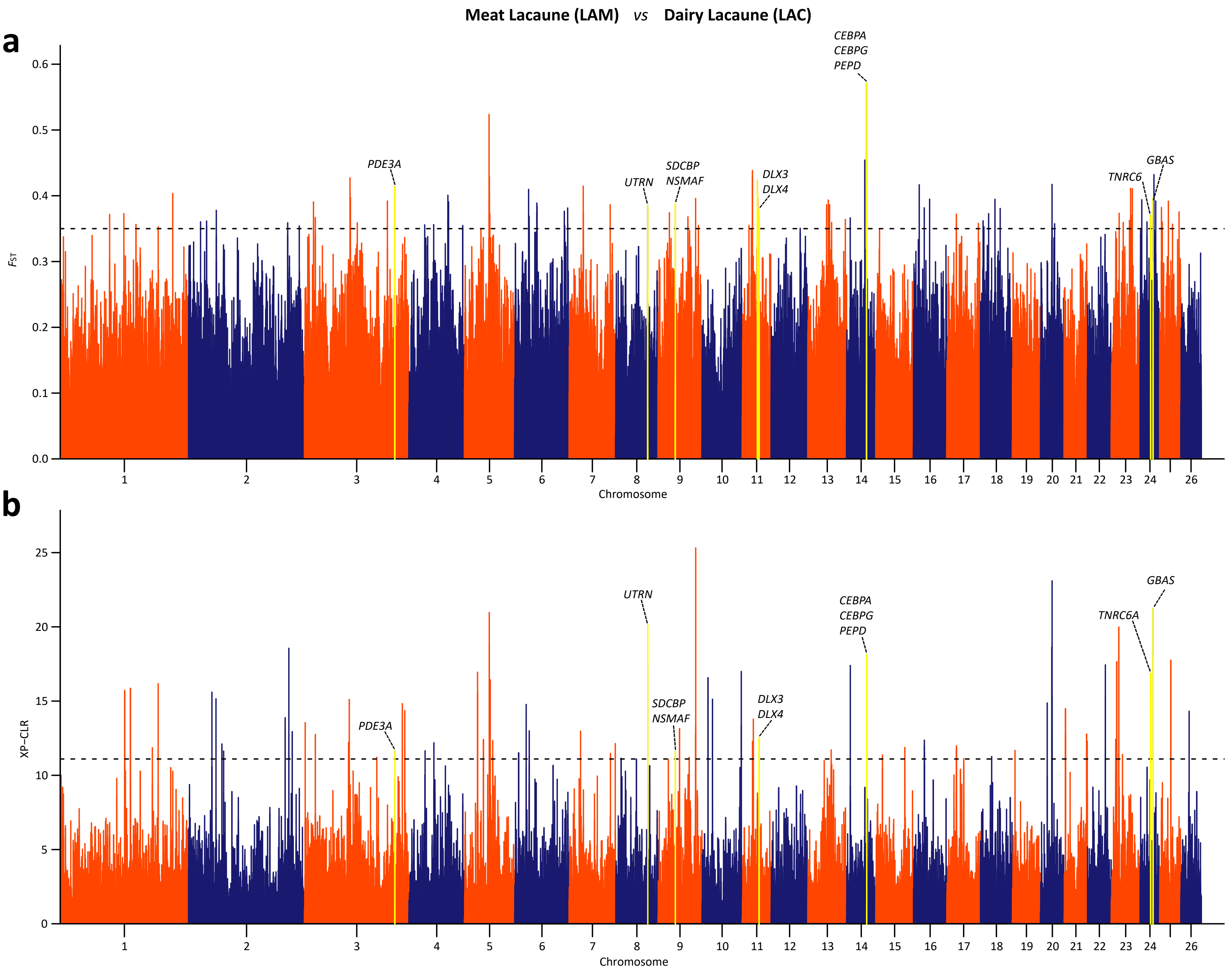

Supplementary Figure 5**.** Manhattan plots of **a**, pairwise *F*ST and **b**, XP-CLR with meat production in the comparison of Meat Lacaune (LAM) and Dairy Lacaune (LAC) breeds. The top 0.02% of the empirical distribution of *F*ST and 0.05% of the XP-CLR scores is indicated by a dotted line.
